# A Rare Case of HELLP Syndrome with Hematomas of Spleen and Liver, Eclampsia, Severe Hypertension and Prolonged Coagulopathy—A Case Report

**DOI:** 10.3390/ijerph19137681

**Published:** 2022-06-23

**Authors:** Małgorzata Lewandowska, Monika Englert-Golon, Zbigniew Krasiński, Paweł Piotr Jagodziński, Stefan Sajdak

**Affiliations:** 1 Department of Biochemistry and Molecular Biology, Poznan University of Medical Sciences, 60-781 Poznan, Poland; pjagodzi@ump.edu.pl; 2 Division of Gynecological Surgery, University Hospital, 33 Polna Str., 60-535 Poznan, Poland; 3 Department of Gynaecology Obstetrics and Gynaecological Oncology, Division of Gynecological Surgery, Poznan University of Medical Sciences, 60-535 Poznan, Poland; m.golon2@wp.pl (M.E.-G.); ssajdak@ump.edu.pl (S.S.); 4 Department of Vascular, Endovascular Surgery, Angiology and Phlebology, Poznan University of Medical Sciences, 61-848 Poznan, Poland; zbigniew.krasinski@gmail.com

**Keywords:** HELLP syndrome, spleen, liver, hematomas, thrombocytopenia, thrombotic microangiopathies, pregnancy

## Abstract

The HELLP syndrome (hemolysis, liver damage and thrombocytopenia) is a rare (0.5–0.9%) but serious complication of pregnancy or puerperium associated with a higher risk of maternal and fetal mortality and morbidity. Liver and spleen hematomas rarely entangle (<2%) HELLP cases, but rupture of the hematomas presents an immediate threat to life. We present the history of a 35-year old pregnant woman (at the 31st week) admitted to our hospital due to the risk of premature delivery. On the first day, the patient did not report any complains, and the only abnormality was thrombocytopenia 106 G/L. However, within several hours, tests showed platelet levels of 40.0 G/L, LDH 2862.0 U/L and AST 2051.6 U/L, and the woman was diagnosed with severe HELLP syndrome, complicated by hematomas of the liver and spleen, seizures (eclampsia), severe arterial hypertension and coagulation disorders. The purpose of this article is to highlight the need for early investigation of the causes of thrombocytopenia and the differentiation of HELLP from other thrombotic microangiopathies (TMAs).

## 1. Introduction

The HELLP syndrome is a rare (0.5–0.9%) but serious complication of pregnancy or puerperium, associated with a higher risk of maternal and fetal mortality and morbidity [1,2,3,4,5,6]. Liver and spleen hematomas rarely entangle (<2%) HELLP cases [7,8], but rupture of the hematomas is a state of immediate threat to life [5,6,9]. Adverse effects in pregnant women with the HELLP syndrome include mainly cerebral bleeding and the development of disseminated intravascular coagulation (DIC), liver and kidney failure as well as heart failure and pulmonary edema [3,10,11,12,13,14,15]. Maternal mortality in HELLP is estimated at 1–25% [3,15]. Perinatal mortality of fetuses of mothers with this syndrome is estimated at 7–34% [15,16], and preterm birth affects about 70% of HELLP cases [17].

The complete form of the HELLP syndrome is characterized by intravascular hemolysis (H), the elevation of liver enzymes (EL) and lower platelet count (LP), including lactate dehydrogenase (LDH) > 600 U/L, aspartate aminotransferase (AST) ≥ 70 U/L and thrombocytopenia < 100.0 G/L respectively [3,18]. Delayed diagnosis of the HELLP syndrome may increase potential maternal and fetal mortality and morbidity.

However, early diagnostic of HELLP can be a challenge: (1) This syndrome most often occurs in conjunction with preeclampsia (PE), a severe form of pregnancy-induced hypertension (PIH), but can occur on its own (in 10–30% of cases) [3,18,19]. Risk factors for HELLP are similar to those for other PIH forms [19,20,21,22]; (2) Prodromal symptoms of HELLP are nonspecific (malaise, nausea, epigastric pain, headache or visual symptoms) [3,23] but these prodromal symptoms may not be present either [2,3]; (3) Importantly, HELLP syndrome belongs to a broad spectrum of thrombotic microangiopathies (TMAs) characterized by hemolytic anemia, thrombocytopenia and organ damage as a result of microclots. Differentiation with other TMAs have important therapeutic implications [16,18,24,25].

This report describes a patient in the 31st gestational week, in whom there was a sudden development of severe HELLP syndrome, complicated by hematomas of the liver and spleen, seizures (eclampsia), severe arterial hypertension and prolonged coagulation disorders. The disease is characterized by rapidly increasing biochemical changes, and the only prodromal symptom was thrombocytopenia (106 G/L). The purpose of this article is to highlight the need for early investigation of thrombocytopenia causes and differentiation of HELLP from other thrombotic microangiopathies (TMAs).

## 2. Case Description

The patient was a 35-year-old pregnant woman in her 31st week of a singleton pregnancy (grav 2, para 1) admitted to the Gynecological and Obstetric Hospital (in Poznan, Poland) due to history of minor lower abdominal pain. The women did not report chronic diseases or diseases during pregnancy, including hypertension or neurological diseases. Pre-pregnancy body mass index (BMI) was 25.6 kg/m^2^, the current BMI was 29.6 kg/m^2^, the arterial pressure was stable at 100–110/60 mmHg, and the heart rate was 72/min.

The examination (including cardiotocography and ultrasound) confirmed intact membranes, no uterine contractions, shortened cervix (the length of which was only 17 mm) as well as normal uterine arteries and umbilical cord flows; the placenta did not show pathologic findings. Due to the risk of premature delivery the patient received betamethasone (to stimulate fetal lung development), magnesium and vaginal progesterone.

On the first day (in hospital) the patient did not report any complaints and arterial pressure was constantly normal. The blood count examination revealed leucocytes 9.88 G/L; hemoglobin 6.90 mmol/L; hematocrit 0.32 L/L; and the only abnormality was the level of platelets 106.0 G/L. Results of tests of urine were correct, without proteinuria.

On the second day, the mother’s blood was sent (in planned mode) to evaluate a wider range of laboratory tests. The condition of the mother (and fetus) was constantly normal. However, during this time, there were short-lasting convulsions (without preemptive clinical symptoms). Diazepam 10 mg (intravenously) was administered. Seizures subsided, and the arterial pressure was at 210/110 mmHg.

The patient was admitted to the Intensive Care Unit (ICU). Treatment, diagnostic and constant monitoring of the mother (and fetus) was continued. At admission to the ICU, the arterial pressure was 210/100 mmHg. Neurological tests showed equal and symmetrical pupils, symmetrical and normal tendon reflexes (++), lack of meningeal and other pathological symptoms. The Glasgow Coma Scale (GSC) was 15 = 4/4 + 5/5 + 6/6. The patient don’t have edema (at the time of hospitalization and transfer to the ICU). Antihypertensive treatment (labetalol, 200 mg in 50 mL of 0.9% NaCl, by intravenous infusion 4–10–2 mL/hour) as well as oxygen therapy were conducted. Anti-convulsant therapy was ordered (magnesium sulfate by intravenous infusion: bolus 4–6 g iv in 20 min, then 1–2 g/h).

The results of the earlier laboratory tests (“Before ICU”) showed a reduced level of platelets (95.0 G/L) and an increased level of AST (81.40 U/L) (Table 1). The HELLP syndrome and eclampsia were diagnosed. The need for blood products was reported. Obstetricians decided to perform a cesarean section after stabilizing the general condition of the patient.

Within the first hour of stay in the ICU, the pressure was stabilized at 150/100 mmHg; however, a convulsive state occurred.

New laboratory tests (sent at admission to the ICU) showed the build-up of abnormalities: platelets 46.0 G/L (manual counts 40.0 G/L); AST 2051.60 U/L; LDH 2862.000 U/L; total bilirubin 22.2 µmol/L (1.30 mg/dL); and d-dimers 31,657.0 ng/mL (Table 1, “At admission”).

The patient was immediately (due to the status epilepticus) intubated, and an emergency caesarean section was performed under general combined anesthesia (Sevoflurane 2 Volume% and N_2_O:Oxygen = 2:1 with a flow of 2 L/min; fentanyl and rocuronium). A boy was born with a weight of 1655 g and Apgar 5, 7, 7 in the 1st, 3rd and 5th minute, respectively. In order to achieve adequate uterine tonus, PABAL (carbetocinum 100 µg) was administered intravenously (the indication for carbetocinum was preterm birth). Intraoperatively, the infusion of labetalol was continued. Intraoperatively, platelet concentrate, red blood cells and fresh frozen plasma were transfused. The laboratory results after cesarean section (C.S.) are presented in Table 1.

Immediately after the operation, a bedside ultrasound scan was performed and a subcapsular hematoma of the spleen of 17 × 52 mm was found. In the liver image, in the central part, numerous small outbreaks of interstitial hematomas in the area of 73 mm in diameter and subcapsular hematoma in the right lobe of 13 mm in diameter were observed.

On the following days, the antihypertensive treatment (of labetalol infusion and single doses of ebrantil), magnesium sulfate infusion, furosemide (intravenously) for brain edema prevention, dexamethasone (intravenously) for improved platelet counts, anticoagulant prophylaxis, antibiotics and albumin were administered. Blood preparations were applied early on. The acid-base and water-electrolyte balance was monitored and equalized, and creatinine levels were normal (Table 1). Arterial pressure was 120/80 mmHg with pressure spikes up to 200/100 mmHg. In chest X-rays, no lung abnormalities were observed.

The results of neurological tests were constantly normal and symptoms of cerebral pathologies were not presented. Therefore, it was decided to not transport the patient for neuroimaging (at that time, a CT scan/resonance was not available at our hospital).

On the first–third day in ICU, the highest deviations of the transaminase and dehydrogenase levels, and the results of blood morphology (despite the absence of bleeding symptoms), as well as the level of d-dimers were found. A low level of antithrombin III (AT-III) (41% and 51%) was found on the second to fourth day (Table 1). Other indicators of coagulology remained within the range of normal.

On the fourth day, normalization of these tests (transaminase, dehydrogenase and blood morphology) was observed (Table 1). On days four to five, arterial pressure stabilization was observed (120/80 mmHg). On the seventh day, in regular ultrasound examinations, only minor outbreaks of interstitial hematomas of the right lobe of the liver were found.

On days 6th–8th, new abnormalities were noted in coagulology: d-dimers were in-creased at 77,229.0 ng/mL and AT-III level was decreased at 51% (with slight changes in other coagulology indicators). The AT-III preparation was administered and intravenous heparin was applied under APTT control. After three days an improvement was achieved and the treatment was continued with enoxaparin (2 × 60 mg) administered subcutaneously.

After the intensified treatment, laboratory tests showed: platelets 189.0 G/L; Hb 6.80 mmol/L; Hct 0.34 L/L; leucocytes 4.97 G/L; LDH 258.0 U/L; ALT 28.9 U/L; AST 24.0 U/L; D-dimers 4 028.0 ng/mL; fibrinogen 4.03 g/L; creatinine 68.1 µmol/L (0.77 mg/dL); total protein 65.4 g/L. On the 16th day after the cesarean section, the patient was discharged from the hospital in good general condition. The newborn was discharged home in good general condition on the 37th day of life.

The basic results of the newborn were as follows: Immediately after giving birth, the features of Respiratory Distress Syndrome (RDS) were diagnosed; Preliminary laboratory tests did not show anemia, thrombocytopenia or leukopenia (however, anemia was found in the 22nd day of life); Neurological disorders have not been reported; In echocardiography, closed ductus arteriosus, correct flows and flaccid atrial septum were found; Abdominal ultrasound showed correct image. Other examinations have included metabolic tests, microbiological tests and ophthalmological consultations.

## 3. Discussion

In this case:

- Thrombocytopenia (PLT) 106 G/L was the first (prodromal) symptom of the disease. The HELLP symptoms develop dynamically in the pregnant women without pre-existing hypertension and proteinuria and other clinical prodromal symptoms.
- Abnormal results (of intravascular hemolysis, thrombocytopenia and features of liver damage) persisted for three days and then improved.
- Urgent caesarean section and multidirectional intensive treatment allowed the mother and child to recover.

Our patient met the diagnostic criteria of complete HELLP syndrome (hemolysis, thrombocytopenia, liver damage), according to the Tennessee classification and the Mississsippi-Triple Class System [3,26].

According to the literature, hemolysis is diagnosed by elevated levels of lactate dehydrogenase LDH > 600 U/L, an increased serum level of bilirubin (total bilirubin ≥ 1.2 mg/dL; 20.5 µmol/L), and a decreased level of haptoglobin. The hemoglobin released from erythrocytes forms complexes with haptoglobin, but when haptoglobin levels are exhausted, the concentration of free hemoglobin (in serum and urine) increases. Although currently less tested, the presence of schistiocytes in the blood smear tests and an increase in the number of reticulocytes, spherocytes and polychromatophilic erythrocytes confirm the hemolysis [18]

However, this case demonstrates that thrombocytopenia in pregnancy urgently requires further diagnosis and our case drew attention to this. The HELLP syndrome must be differentiated from other thrombotic microangiopathies (TMAs) characterized by hemolysis (and microangiopathic hemolytic anemia), thrombocytopenia and damage to various end organs (especially kidneys) due to endothelial damage and microclots [18,24,25].

Classically, the pathogenesis of HELLP is believed to fall within the pathogenesis spectrum of preeclampsia and includes abnormal first trimester placenta development, endothelial dysfunction and vasoactive release (with rising blood pressure) [2,26,27]. However, the role of the immune response including the role of the complement system is also emphasized [28,29]. A dysfunctional damaged endothelium and constricted blood vessels cause the breakdown of erythrocytes (hemolysis and hemolytic anemia). Blood clots occur in the tiny blood vessels (platelets aggregate), and the result is organ damage. In the HELLP syndrome, the liver is the major organ damaged, but the kidneys are often (>50% of HELLP cases) damaged as well [18,25,26,30,31]. It is possible that endovascular deposition of fibrin deposits (not connected with disseminated intravascular coagulation, DIC) limits blood flow through the liver sinusoids, and hypoxia leads to the formation of focal infarcts, subcapsular hematomas and interstitial hemorrhage [7,9,14,32]. Importantly, pre-existing preeclampsia (hypertension and proteinuria) is not found in 10–30% of HELLP cases [3,18].

The HELLP syndrome must be differentiated from TMAs, such as: thrombotic thrombocytopenic purpura (TTP); typical hemolytic uremic syndrome (HUS); and atypical hemolytic uremic syndrome (a-HUS) [24,25,26].

(1) Thrombotic thrombocytopenic purpura (TTP) results from an acquired or (less frequently) congenital deficit of ADAMTS13 protease (a disintegrin and metalloproteinase with a thrombospondin type 1 motif, member 13). Deficiency of ADAMTS13 results in the formation of unusually large activated multimers of von Willebrand factor (VWF) on endothelial cells and the aggregation of platelets. This leads to blood clots in the small vessels and to hemolysis [18,24,25,33].
(2) Typical hemolytic uremic syndrome (HUS) is caused by a bacterial infection (mainly Escherichia coli) producing the Shiga toxin (and is abbreviated as STEC-HUS). Shiga toxin damages the endothelium; it leads to hemolytic anemia, thrombocytopenia and kidney damage. STEC-HUS occurs mainly in children but can occur in adults, and diagnosis requires the identification of the toxin or the toxin-producing bacteria [24,25,33].
(3) Atypical hemolytic uremic syndrome (a-HUS) is a rare and life-threatening disease that affects children and adults. A-HUS is the result of genetic (40–60% of a-HUS cases) or acquired disturbances in the regulation (deficiencies in the regulators) of the alternative complement pathway. Increased production of complement-C5 terminal components (C5a and C5b/C5b-9) is reported in a-HUS. Non-genetic a-HUS triggers include, for example, pregnancy as well as infections, inflammations, autoimmune diseases, surgeries, transplants and drugs [16,25,33,34,35,36,37]. In particular, pregnancy can trigger Pregnancy-a-HUS [37], which is found very rarely (1 in 25,000 pregnancies) [16,34,35,38]. Importantly, there is increasing evidence of increased complement activation in women with HELLP syndrome [25,26,30,31].

To sum up, thrombocytopenia (platelet count < 150 G/L) affects about 10% of pregnant women, and severe cases of thrombocytopenia in pregnancy are associated with the TMAs listed above or with immune thrombocytopenia (ITP). Gestational thrombocytopenia (GT) is a mild condition and is the most common form of thrombocytopenia in pregnant women (affects 70–80% of cases), which may reduce our vigilance. Differentiation of thrombocytopenia in pregnancy (and limitations in this regard) has been extensively described in the literature [39,40,41,42,43,44]. In GT, platelet count is rarely below 100 G/L; other possible etiologies should be urgently excluded if this level is below 50 G/L; and if thrombocytopenia persists for more than one to two months to delivery, the differential diagnosis should be extended [39,44]. Low platelet count concerns TTP, HELLP, and a-HUS, but severe thrombocytopenia (especially <30 G/L) is associated primarily with TTP [18]. Severe ADAMTS13 protease deficiency (<5%) differentiates TTP from HELLP and a-HUS [18,33,37]. HELLP is associated with a characteristic coagulology; prothrombin time (PT) and activated partial thromboplastin time (APTT) are initially within the normal values, but the level of fibrin degradation products (D-dimers) and thrombin complexes with antithrombin-III increases, and the level of antithrombin-III (AT-III) activity decreases [25]. The diagnosis of a-HUS can be favored, among others, by high LDH to AST ratio (>10 to 1) suggesting significantly higher blood cell hemolysis compared to hepatitis [18]. To confirm a-HUS, tests (including genetic tests) are performed for the components of the complement system [16,25,34,35,36,37].

Importantly, however, there is a growing body of evidence for the role of complement activation in the pathogenesis of HELLP [25,30,31,45]. HELLP and a-HUS display a strong similarity, demonstrated, among others, by Vaught et al. [26]. In HELLP and a-HUS, elevated levels of C5a and C5b-9, as well as common gene mutations of the regulators of the alternative complement pathway were found [16,24,26].

This potential similarity between HELLP and a-HUS have important therapeutic implications. The drug approved for the treatment of a-HUS is eculizumab (a humanized monoclonal antibody), which inhibits the cleavage of the C5 complement component into C5a and C5b, which reduces endothelial disturbances [24,25,33]. The European Medicines Agency (EMA) and the American Food and Drug Administration (FDA) have approved the use of eculizumab in the treatment of a-HUS and paroxysmal nocturnal hemoglobinuria (PNH) [16]. There is increasing evidence of the beneficial effects of eculizumab in pregnant women with a-HUS (and PNH); in many cases, the mother’s kidney function was protected and pregnancy was extended by several weeks [16,25,31].

Importantly, the number of publications in which off-label use of eculizumab in pregnant women (in HELLP syndrome and preeclampsia) was reported is increasing. Reduced maternal morbidity (especially associated with renal failure) and prolonged pregnancy was achieved [16,24,31]. Unfortunately, there are no complete data on the efficacy and safety of eculizumab in off-label indications in pregnant women yet [16,34]. Further research is being conducted [16,25,35].

The need to look for new diagnostic markers for HELLP should also be emphasized. Beyond the modified Ham test evaluated by Vaught et al. (as below) [26], markers such as the sFlt-1/PlGF (i.e., fms-like tyrosine kinase 1 to placental growth factor ratio) as well as other anti- and pro-angiogenic proteins or biomarkers were examinated. However, none of the new markers have been approved [3].

According to the literature, clinical symptoms of hemolysis and thrombocytopenia in HELLP usually disappear after about three days [24]. If the disease lasts longer, the differential diagnosis is to be extended and alternative treatment should be considered [24].

Classically, the basic therapy of HELLP syndrome is maintenance treatment and childbirth, which interrupts the cascade of disorders leading to the development of this disease [3]. In the case of unruptured hematomas (of liver and spleen), conservative treatment is recommended [9,32]. The treatment may require the use of antihypertensive drugs, magnesium sulfate infusion, blood products, immunoglobulins, steroids, anticoagulants and plasma exchange [3,7,25,46,47,48,49,50,51]. In any case, one should avoid injuries that may be caused by, for example, convulsions or abdominal palpation but also by the transport of the patient. Continuous monitoring of the status of the fetus and mother is necessary.

The use of steroids in HELLP is one of the main controversies; however, there are no prospective randomized studies on this [3]. Glucocorticoids may improve platelet counts [3,48,49]. In HELLP treatment, results obtained by James et al. or others showed a positive effect of steroids on reducing perinatal mortality, although this effect has not been demonstrated in studies by Svenningsen et al. and Fonseca et al. [3]. For the treatment of other causes of thrombocytopenia, the first-line treatment recommended for immune thrombocytopenia (ITP) includes corticosteroids as well as intravenous immunoglobulin therapy. In a-HUS, initial management includes immediate plasma exchange (PE) (pending the results of the target diagnostics); steroids can also be used, and renal replacement therapy may be needed, and Eculizumab halts the complement cascade. Treatments for TTP include plasmapheresis (e.g., cryosupernatant plasma-based) and platelet transfusion [39]. Gestational thrombocytopenia does not respond to corticosteroids, and two mechanisms of this thrombocytopenia are taken into account: hemodilution and accelerated platelet clearance [39,44].

## 4. Conclusions

This case description is to serve as a reminder that thrombocytopenia in pregnant women urgently requires further diagnosis (and differentiating from other thrombotic microangiopathies) and may be the first symptom of HELLP syndrome. HELLP may develop dynamically in pregnant women without pre-existing hypertension and proteinuria, and without previous other prodromal clinical symptoms. An extension of diagnosis and treatment is necessary when disorders persist for more than three days.

Because the correlations between HELLP severity and clinical symptoms (e.g., ar-terial hypertension) are not conclusive, there is a need to implement standards for diagnostic criteria and treatment options.

Increasing the amount of evidence may help establish criteria, procedures, and guidelines for a treatment with complement inhibitors (such as eculizumab) in pregnant women with HELLP.

## Figures and Tables

**Table 1:** The laboratory results during intensive treatment of the HELLP case.

		The Laboratory Results during Intensive Treatment of the HELLP Case					
Tests [Units]	(Norms)	Before ICU	ICU-1. Day at Admission	ICU-1/2. Day 2–5 h after C.S.	ICU-2/3. Day	ICU-4. Day	ICU-6. Day
Platelets [G/L]	(150–400)	95.0	40.0 *	70.0 *–65.0 *	68.0 *	118.0	86.0 *
Hb [mmol/L]	(6.95–9.75)	7.10	8.00	6.00–6.00	5.90	7.30	7.20
Hct [L/L]	(0.34–0.51)	0.33	0.38	0.28 0.27	0.27	0.35	0.34
Leucocytes [G/L]	(3.98–10.04)	10.62	11.39	9.74–11.80	12.35	13.48	12.26
Total Bilirubin [mg/dL]	(<1.2)	0.18	1.30	0.74	0.46	0.59	0.33
[µmol/L]	(<20.5)	3.1	22.2	12.7	7.9	10.1	5.6
LDH [U/L]	(135–214)	-	2862.00	1222.50	1825.00–2267.00	729.0	386.0
AST [U/L]	(<32.0)	81.4	2051.60	1855.00	1328.50–1903.50	571.9	40.50
ALT [U/L]	(<33.0)	35.10	532.70	421.80	578.0–816.30	621.00	133.90
CRP [mg/L]	(<5.0)	0.49	26.36	78.11	115. 38	55.33	36.20
Procalcitonin [ng/mL]	(0.5–2.0)	-	1.87	2.26	1.89	0.95	0.23
AT III [%]	(83–128%)	-	-	-	41–71%	51–83%	84%
D-dimer [ng/mL]	(0.0–550.0)	1290.0	31,657.0	23,656.0–21,672.0	22,577.0	3479.0	77,229.0
Fibrynogen [g/L]	(2.00–3.93)	3.18	2.00	1.55	3.40	4.69	3.31
Creatinine [mg/dL]	(0.50–0.90)	-	1.00	0.89	0.89	0.86	0.66
Creatinine [µmol/L]	(44.2–79.6)	-	88.4	78.7	78.7	76.0	58.3
Total Protein [g/L]	(66.0–88.0)	-	66.0	50.3	-	44.8	47.3
Electrolytes **							
Mg^2^^+^ [mmol/L]	(0.66–1.07)	-	0.73	0.77	0.81	-	0.74
Na^+^ [mmol/L]	(135.0–145.0))	139.0	136.0	138.0	139.0	141.0	141.0
K^+^ [mmol/L]	(3.5–5.5)	4.28	4.09	4.65	4.56	4.24	4.29
Cl^−^ [mmol/L]	(95.0–107.0)	102.7	91.7	99.0	101.3	99.9	100.9
Ca^2+^ [mmol/L]	(2.15–2.50)	-	2.33	2.13	2.09	-	2.16

* manual counts; ** Mg^2^, Na^+^, K^+^, Cl^−^, Ca^2+^: magnesium, sodium, potassium, chloride, calcium (serum concentration) respectively. ALT: alanine aminotransferase; AST: aspartate aminotransferase; AT: antithrombin; CRP: C-reactive protein; C.S.: cesarean section; Hb: hemoglobin, Hct: hematocrit; LDH: lactate dehydrogenase.

## Data Availability

The data presented in this study are available on request from the corresponding author.

## Abbreviations

ADAMTS13	A disintegrin and metalloproteinase with a thrombospondin type 1 motif, member 13
a-HUS	Atypical hemolytic uremic syndrome
ALT	Alanine aminotransferase
APTT	Activated partial thromboplastin time
AST	Aspartate aminotransferase
AT	Antithrombin
BMI	Body mass index
Ca	Calcium
Cl	Chloride
CRP	C-reactive protein
EMA	European Medicines Agency
FDA	(American) Food and Drug Administration
GT	Gestational thrombocytopenia
Hb	Hemoglobin
Hct	Hematocrit
HELLP	Hemolysis (H), elevation of liver enzymes (EL) and low level of platelets (LP)
HUS	Hemolytic uremic syndrome
ICU	Intensive care unit
ITP	Immune thrombocytopenia
K	Potassium
LDH	Lactate dehydrogenase
Mg	Magnesium
Na	Sodium
PE	Preeclampsia
PIH	Pregnancy-induced hypertension
STEC-HUS	Hemolytic uremic syndrome caused by *Escherichia coli* infection producing Shiga toxin
TMA(s)	Thrombotic microangiopathies
TTP	Thrombotic thrombocytopenic purpura (TTP)
